# New perspectives on the physiological basis of muscle loss

**DOI:** 10.1113/EP092565

**Published:** 2026-02-16

**Authors:** Alistair J. Monteyne, Marlou L. Dirks

**Affiliations:** ^1^ Public Health and Sport Sciences, Faculty of Health and Life Sciences University of Exeter Exeter UK; ^2^ Human and Animal Physiology Wageningen University Wageningen The Netherlands

## 1 INTRODUCTION

This special issue of *Experimental Physiology* presents a collection of papers arising from The Physiological Society conference, ‘New perspectives on the physiological basis of muscle loss’, hosted by the University of Exeter in September 2024. The 2‐day meeting brought together speakers of all career stages, from early‐career researchers to leading authorities in their field, from across Europe and further afield, allowing a blend of cutting‐edge research and career insights. Its intimate and focused format fostered a convivial yet scholarly environment that encouraged rigorous scientific exchange. In this Editorial, we outline the origins of the conference and highlight how each article in this special issue advances our understanding of the physiological basis of skeletal muscle loss.

## 2 MEETING ORIGINS

The meeting originated from a desire to explore contemporary perspectives on the underlying causes of muscle loss in the context of ageing, disuse, inactivity and malnutrition, whilst also exploring intervention strategies to mitigate such losses. An underlying message that resonated throughout the meeting was just how pervasive and pressing the issue of muscle loss is in an ageing and ailing society.

The meeting featured evidence spanning the full spectrum of research, from highly mechanistic insights, through experimental physiology, to emerging clinical applications. Equally valuable were the detailed discussions on methodologies for assessing muscle protein turnover, during which several long‐standing assumptions were examined and challenged, in granular detail. Although protein turnover featured prominently throughout the meeting, the scientific dialogue was by no means limited to this topic and also encompassed advances in extracellular vesicles, oligonucleotide‐based senotherapeutics and the role of hydrogen peroxide in cellular function. The invited talks were complemented by excellent original data presentations from early‐career researchers and by ample opportunity for informal discussions, which fostered an environment of candid, thoughtful and productive debate. The outputs of this meeting are presented herein, with articles that span methods and mechanisms across experimental physiology.

## 3 MECHANISTIC PHYSIOLOGY

In this Special Issue, Kuznetsova and Hood (2026) review how mitophagy, the selective tagging and removal of dysfunctional mitochondria and an imperative element of maintaining the mitochondrial pool, plays a potentially crucial role in modulating muscle health. The review highlights recent advances made in the measurement of mitophagy within skeletal muscle and the emerging proteolytic role of the lysosome in facilitating the degradation of dysfunctional mitochondria. The distinct effects of age, exercise training and disuse on mitophagy and lysosomal adaptations are discussed at length. The authors highlight the emerging nature of the research, particularly concerning the terminal steps of organelle degradation mediated via lysosomes.

In an authoritative review, Professor Malcolm Jackson follows on from his presentation at the meeting by reframing reactive oxygen species (ROS), such as hydrogen peroxide, as essential signalling molecules in skeletal muscle plasticity, whilst providing insight into the experimental models from which recent findings are derived (Jackson, 2026). He highlights the central role of peroxiredoxins as initiators of transcription and, therefore, potential mediators of adaptation and age‐related muscle loss. The evidence reviewed supports the concept that disrupted redox signalling contributes to the decline in skeletal muscle mass and function with age. Specifically, that ageing is associated with increased mitochondrial ROS production, altered antioxidant enzyme activity and impaired redox‐sensitive responses to contractile activity, which ameliorates the muscle's capacity to adapt to muscle contraction, which may in turn contribute to the progression of sarcopenia. What emerges is a complex picture of redox regulation in ageing muscle, which suggests that therapeutic strategies aimed at restoring redox signalling may be effective in facilitating healthy muscle ageing and ameliorating sarcopenia.

As with the prior two articles, Ginevičienė and colleagues (2026) also focus on sarcopenia. They conducted a multi‐omics investigation of sarcopenia and frailty in an elderly Lithuanian population of 204 participants, 122 of which were diagnosed with sarcopenia and/or frailty and 82 who were healthy, community‐dwelling older adults. They sought to identify how genetic variation, epigenetic modifications and telomere length contribute to sarcopenia and frailty‐related traits in older adults. In line with the established benefits of possessing an adequacy of muscle mass, lean mass index correlated negatively with comorbidities and positively with performance. The authors identified two genetic variants that were significantly associated with handgrip strength and 12 single‐nucleotide polymorphisms that were associated with lean mass. Moreover, differentially methylated sites in immune‐related genes were linked to lean mass, and shorter telomere length was associated with pronounced sarcopenia and frailty. Such data may begin to explain individual variation in sarcopenic progression and offer a pathway for precision medicine strategies aimed at preserving musculoskeletal health. They may also offer novel genetic and epigenetic biomarkers for early diagnosis and treatment.

In the final contribution to this special issue, Rosa‐Caldwell and colleagues (2026) utilise an elegant animal model to investigate mitochondrial health in the context of a cycle of weight loss and regain, as a means of exploring the effect of anorexia nervosa, a psychiatric disorder characterised by prolonged caloric restriction and concomitant skeletal muscle atrophy. In their experiment, Sprague–Dawley rats underwent a 30‐day simulated anorexia protocol, followed by varying durations of weight regain, via ad libitum feeding. They observed reduced muscle performance, which was associated with reduced reliance on mitochondrial complex I and genes related to mitochondrial quality control. Intriguingly, many of these adaptations persist even after short‐term weight recovery, although longer‐term, sustained weight regain normalised several facets of mitochondrial health, with the exception of mitochondrial fission. Nonetheless, the authors note that there still may be long‐term consequences to mitochondrial dynamics.

## 4 METHODOLOGY

The methodological nuances and interpretative challenges of deuterium oxide (D_2_O)‐based approaches to measure skeletal muscle protein turnover, particularly the difficulty in reconciling findings from rodent models to humans, are critically examined by Lim and colleagues (2026). They effectively lay out the merits and pitfalls of both full proteome and fractional turnover approaches, and advocate for their co‐existence to accurately capture the effects of loading, ageing, disuse and nutritional interventions on muscle protein dynamics. This article offers an invaluable perspective for any reader wishing to utilise or interpret deuterium oxide‐based measurements of protein turnover.

Prokopidis and colleagues (2026) also address methodological considerations when measuring muscle protein turnover, presenting comprehensive data on how several different commonly utilised experimental models of uncomplicated disuse (bed rest, unilateral lower limb immobilisation and head‐down tilt) affect muscle protein synthesis (MPS) rates in healthy adults. They synthesise data from four studies examining the impact of bed rest, nine examining the impact of unilateral lower limb immobilisation and three the impact of 6° head‐down tilt, with the latter excluded from the meta‐analysis due to the low sample size. Their analysis revealed a pronounced and consistent suppression of MPS across conditions, with bed rest reducing MPS by an average of −0.017%/h and lower limb immobilisation by −0.015%/h. Given the comparable magnitude of suppression in MPS induced by bed rest and lower‐limb immobilisation, the authors maintain that future studies of disuse‐related muscle loss can select a model of uncomplicated disuse based on practical and scientific considerations, rather than the assumption that one approach represents a more potent catabolic stimulus than the other.

Van de Meene and colleagues (2026) present a useful technical review of the use of electrical pulse stimulation (EPS) in murine C2C12 studies, where wide variability in EPS protocols may be hampering both reproducibility and the ability to compare study findings. They analysed 54 peer‐reviewed studies that employed EPS in C2C12 cells, observing largely consistent stimulation frequency, but significant variation in voltage and pulse duration. They note a range of endpoints that can be measured using this model, with glucose uptake the most common. Given that these methods are increasingly used to model exercise in vitro, the findings emphasise the need for more deliberate and judicious protocol design to better match in vivo exercise modalities (e.g. endurance and resistance exercise). The authors provide a useful resource for those wishing to tailor EPS parameters to investigate specific physiological processes and encourage the standardisation of protocols to improve translational and comparability.

## 5 CLINICAL APPLICATION

Our understanding of ventilator‐induced diaphragm dysfunction (VIDD) following mechanical ventilation (MV) is increased by Mesquita and coworkers (2026), who draw on well‐established principles from limb muscle disuse. This approach is particularly compelling in the context of ageing, where older MV patients are more susceptible to diaphragm dysfunction and have poorer survival after MV, with studies directly examining how ageing influences VIDD and subsequent recovery remain scarce. The review summarises current evidence on the effects of MV on the diaphragm, including that of ageing and poses this alongside the more extensive literature on limb muscle disuse, where aged muscles show distinct mechanisms of atrophy and markedly impaired recovery. By drawing these parallels, the authors demonstrate that similar age‐dependent limitations in recovery may exist in the diaphragm. They conclude that only by delineating how adult and aged diaphragms differ in their response to and recovery from MV will we be able to develop therapies to improve the recovery and survival of older patients subjected to MV.

Libramento and colleagues (2026) also take a clinical perspective, reviewing the current understanding of muscle wasting in cancer cachexia, detailing the interplay between inflammation and proteolytic pathways that drive a progressive loss of skeletal muscle mass and function. They highlight emerging evidence that exercise, both aerobic and resistance training modalities, can counter losses of muscle mass and function by attenuating protein degradation. Indeed, although the precise molecular mechanisms through which exercise influences the two primary proteolytic pathways, the autophagy–lysosome and ubiquitin–proteasome systems, remain to be clarified, the authors argue that targeted exercise interventions hold substantial therapeutic promise and should be a focus of future research to improve outcomes for patients with cancer‐associated cachexia.

## 6 SUMMARY

This special issue of *Experimental Physiology*, originating from The Physiological Society meeting ‘New perspectives on the physiological basis of muscle loss’, brings together a diverse and timely body of work that advances our understanding of skeletal muscle loss across ageing, disuse, disease and malnutrition. The articles included herein span a broad swathe of scientific ground, from nuanced considerations when choosing and using specific methods, to contemporary insights for clinical populations, mechanistic reviews and original data. Several contributing authors highlight the need to refine experimental models and methods (Libramento et al., 2026; Lim et al., 2026; Mesquita et al., 2026; van de Meene et al., 2026), whilst the importance and utility of exercise is clearly discernible (Jackson, 2026; Kuznyetsova & Hood, 2026; Libramento et al., 2026; van de Meene et al., 2026). Collectively, the scholarship within this issue demonstrates the multifaceted causes and consequences of muscle loss and the resultant need to develop bespoke interventions to counter such losses in an ageing and increasingly clinically compromised population. We look forward to further discussing many of the themes contained herein, most notably age‐related muscle loss and the physiological mechanisms regulating protein turnover, during a planned follow‐up Physiological Society meeting, ‘Recent advances in nutritional physiology: a muscle‐centric perspective’ hosted at the University of Exeter on 25 and 26 August 2026.

## AUTHOR CONTRIBUTIONS

Alistair J. Monteyne and Marlou L. Dirks wrote the manuscript, contributing equally to its development. Both authors have read and approved the final version of this manuscript and agree to be accountable for all aspects of the work in ensuring that questions related to the accuracy or integrity of any part of the work are appropriately investigated and resolved. All persons designated as authors qualify for authorship, and all those who qualify for authorship are listed.

## CONFLICT OF INTEREST

None declared.

## FUNDING INFORMATION

None.
